# SAR of Sponge-Inspired Hemibastadin Congeners Inhibiting Blue Mussel PhenolOxidase

**DOI:** 10.3390/md13053061

**Published:** 2015-05-15

**Authors:** Hendrik Niemann, Jens Hagenow, Mi-Young Chung, Claire Hellio, Horst Weber, Peter Proksch

**Affiliations:** 1 Institute of Pharmaceutical Biology and Biotechnology, Heinrich-Heine-University Düsseldorf, Universitätsstrasse 1, Geb. 26.23, 40225 Düsseldorf, Germany; E-Mails: hendrik.niemann@hhu.de (H.N.); jens.hagenow@hhu.de (J.H.); mi-young.chung@hhu.de (M.-Y.C.); horst.weber@hhu.de (H.W.); 2 LEMAR UMR 6539 UBO CNRS Ifremer IRD, European Institute of Marine Studies (IUEM), Université de Bretagne Occidentale (UBO), European University of Brittany (UEB), Technopole Brest-Iroise, 29280 Plouzané, France; E-Mail: Claire.Hellio@univ-brest.fr; 3 Biodimar, Université de Bretagne Occidentale (UBO), European University of Brittany (UEB), 6 Avenue Victor Le Gorgeu, CS93837, 29238 Brest cedex 3, France

**Keywords:** antifouling, hemibastadin, phenoloxidase, sponges, copper, *Mytilus edulis*

## Abstract

Hemibastadin derivatives, including the synthetically-derived 5,5′-dibromohemibastadin-1 (DBHB), are potent inhibitors of blue mussel phenoloxidase (PO), which is a key enzyme involved in the firm attachment of this invertebrate to substrates and, thus, a promising molecular target for anti-fouling research. For a systematic investigation of the enzyme inhibitory activity of hemibastadin derivatives, we have synthesized nine new congeners, which feature structural variations of the DBHB core structure. These structural modifications include, e.g., different halogen substituents present at the aromatic rings, different amine moieties linked to the (*E*)-2-(hydroxyimino)-3-(4-hydroxyphenyl)propionic acid, the presence of free *vs.* substituted aromatic hydroxyl groups and a free *vs.* methylated oxime group. All compounds were tested for their inhibitory activity towards the target enzyme *in vitro*, and IC_50_ values were calculated. Derivatives, which structurally closely resemble sponge-derived hemibastadins, revealed superior enzyme inhibitory properties *vs.* congeners featuring structural moieties that are absent in the respective natural products. This study suggests that natural selection has yielded structurally-optimized antifouling compounds.

## 1. Introduction

The world-wide ban of tributyltin (TBT) as an effective, but highly-toxic constituent of anti-fouling (AF) paints in 2008 has spurred the search for eco-friendly alternatives. Currently used compositions of antifouling paints, which are primarily based on copper in addition to so-called booster biocides, like cybutryne (Irgarol^®^), 3-(3,4-dichlorophenyl)-1,1-dimethylurea (Diuron^®^), zinc pyrithione (ZnPT), copper pyrithionine (CuPT) and chlorothalonil, have shown similar or even more severe toxic properties than TBT [1]. Therefore, also for these latter formulations, restrictions of usage, as already decided by the U.S. senate in 2011, will confine their use for maritime industries in the future. Washington became recently the first U.S. state to ban copper-based paints (containing more than 0.5% copper) from 2020 for boats less than 20 m [2]. It is thus urgent to develop new eco-friendly antifouling solutions using innovative concepts, such as biomimetic approaches and the use of compounds with high target specificity, but low general toxicity. Among invertebrate epibionts, blue mussels (*Mytilus edulis*) are considered to be one of the major macrofouling organisms that are known to readily settle on any kind of submerged, hard surface, such as ship hulls, cages used for aquaculture and others. Substrate attachment of *M. edulis* is established through adhesive plaques connected to a byssus stem. The formation of these plaques is catalyzed by a copper-depending phenol oxidase (PO) (E.C. 1.14.18.1), which oxidizes phenolic residues, such as tyrosine, to catechols, like 3,4-dihydroxy-l-phenylalanin (l-DOPA). The catechols are then further converted to *O*-quinones, which are present in so-called *M. edulis* foot proteins (Mefps) [3]. The redox-chemistry of l-DOPA mainly affects the formation of molecular networks within Mefps [4]. Being highly reactive chemical species, these *O*-quinone-bearing scleroproteins easily form intermolecular covalent cross-links with bionucleophils [5]. Until now, ten Mefps have been identified, while for Mefp-1, a firm attachment to substrates, like glass, plastic, wood, concrete and even Teflon^®^, has been shown [6].

In previous studies, sponge-derived hemibastadin derivatives and, in particular, their synthetic analogues, such as 5,5′-dibromohemibastadin-1 (DBHB) (**1**), demonstrated significant antifouling, but low/no general toxic properties towards marine invertebrates, thus highlighting these compounds as promising candidates for future anti-fouling applications [7,8]. DBHB (**1**) was subsequently shown to inhibit blue mussel PO *in vitro*, and the first structure-activity relationships for this compound and structurally related derivatives were reported [8]. It was shown that the α-hydroxyimino-amide moiety of hemibastadins represents an important pharmacophoric substructure of **1**, which is responsible for the strong copper-chelating properties of this compound, thereby presumably causing enzyme inhibition. Furthermore, the presence of bromine substituents at the phenolic rings of **1** increased the enzyme inhibitory properties. We have now synthesized a set of further hemibastadin analogues featuring structural modifications with regard to the substitution pattern of the aromatic rings, the amine substituents and the oxime group. All compounds were then analyzed for their inhibitory activity against blue mussel PO, thereby allowing more detailed predictions on important structural features for future hemibastadin-derived antifouling candidates.

## 2. Results and Discussion

**Figure 1:**
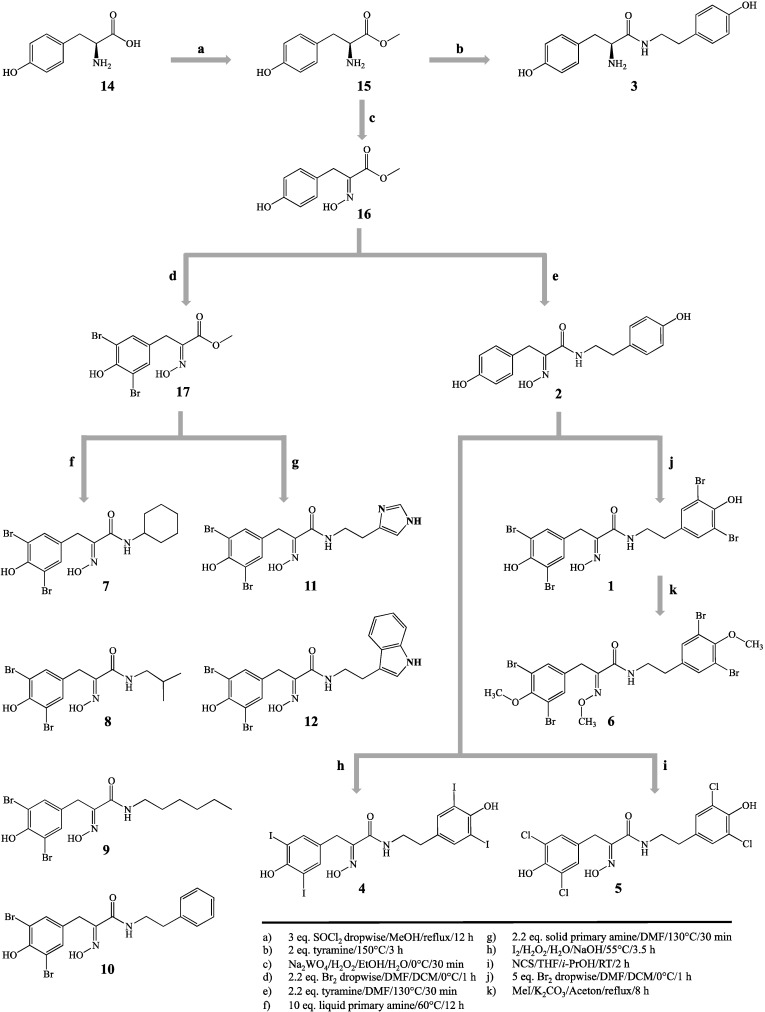
Synthetic approaches to the hemibastadin derivatives reported in this study.

**Figure 2:**
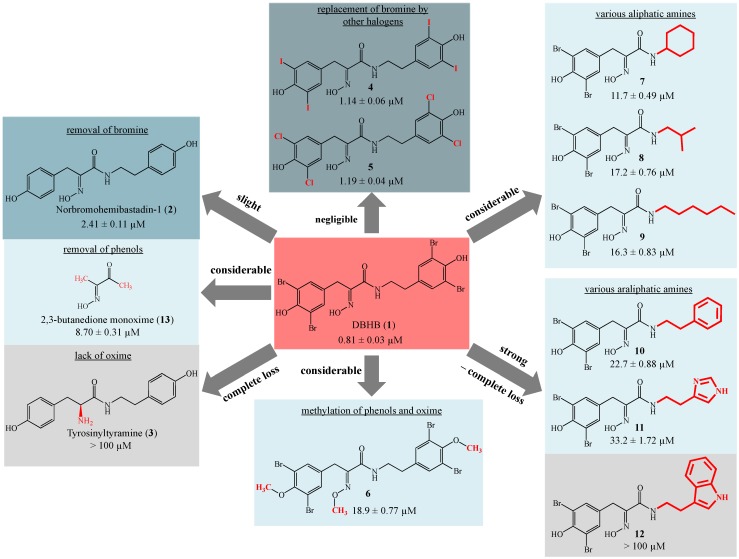
Inhibition of blue mussel phenoloxidase (PO) by 5,5′-dibromohemibastadin-1 (DBHB) (**1**) and its analogues as indicated by their IC_50_ values. Data for Compounds **2**, **3** and **13** were derived from a previous investigation [8].

Hemibastadins consist of a brominated tyrosine moiety featuring an oxime function instead of the amino group and a likewise brominated tyramine unit linked to tyrosine through an amide bond. These compounds are the putative biogenetic building blocks of the more complex bastadins, all of them being typical secondary metabolites of the pacific elephant ear sponge (*Ianthella basta*). The synthetically-derived DBHB (**1**) was reported by our group as one of the strongest inhibitors of blue mussel PO known so far [8]. The synthetic hemibastadin analogues reported in this study (Figure 1) include several structural variations of the parent compound DBHB (**1**). In detail, we replaced the bromine atoms of the former by further halogen atoms, such as chlorine or iodine (**4**,**5**), methylated the phenolic hydroxyl groups, as well as the oxime function (**6**) and substituted the tyramine subunit of DBHB (**1**) by other acyclic or cyclic (including aromatic) amine substituents (**7**–**12**). In addition to these, newly-generated analogues of **1**, norbomohemibastadin-1 (**2**) and tyrosinyltyramine (**3**) that were available from our previous investigation [8] were included in this comparative study on PO inhibition (Figure 2). Analysis of this larger set of compounds suggested the following central structure-activity-relationship (SAR) statements:

Replacement of the bromine atoms of DBHB (**1**) by either chlorine (**4**) or iodine (**5**) resulted only in a negligible reduction of the inhibitory activity of the resulting analogues with very similar IC_50_ values of **4** (1.14 µM) and **5** (1.19 µM) compared to that of **1** (0.81 µM) (Figure 2). Complete loss of halogen substituents as present in norbromohemibastadin-1 (**2**) had a slightly more pronounced effect on the activity (IC_50_ 2.41 µM), indicating that the presence of bulky halogen atoms ortho to the phenolic hydroxyl groups increases the inhibitory activity, albeit only to a small extent. The actual size of the halogen substituents seems to be less important, as indicated by the very similar IC_50_ values of the chlorinated, brominated and iodinated analogues (**1**,**4**,**5**) of **2** (Figure 2).

Substitution of the tyramine unit of DBHB (**1**) by other aliphatic amines (**7**–**9**) had a clearly stronger effect and resulted in a considerable decrease of enzyme inhibitory activity of the different analogues when compared to DBHB (**1**) (Figure 2). The decline in inhibition of congeners bearing other araliphatic amines (**10**–**12**) was even more severe. The presence of the bulky and electron-rich tryptamine moiety *vs.* tyramine as present in Compound **12** resulted in a complete loss of activity. The importance of the p-hydroxyl function of brominated tyramine for the enzyme inhibitory activity of hemibastadin derivatives is apparent upon comparison of **1** with Compound **10**, which exhibits a phenylethylamine moiety instead of tyramine, which causes a strong reduction of inhibitory activity (Figure 2).

It was shown previously that the amine moiety is not an essential structural element that is required for the inhibition of blue mussel PO, as the small synthetic compound 2,3-butanedione monoxime (**13**) that features the alpha-oxo oxime group of the hemibastadins is likewise an enzyme inhibitor [8]. Nevertheless, one may hypothesize that the presence of two phenolic rings in the more active norbromohemibastadin-1 (**2**) with an IC_50_ of 2.41 µM compared to 8.70 µM for **13** [8] provides a better fit of the inhibitor to the enzyme and/or is involved in the stabilization of the enzyme-inhibitor complex.

Methylation of both the oxime moiety and the phenolic hydroxyl groups of DBHB (**1**) caused a more than twenty-fold reduction of the enzyme inhibitory activity of **6** compared to the parent compound **1** (Figure 2).

However, methylation of the oxime hydroxyl group does not have a detrimental effect on the inhibitory activity, as is the case of tyrosinyltyramine (**3**), the latter being completely inactive with regard to the inhibition of blue mussel PO [8]. The alpha-oxo oxime substructure that is shared by the hemibastadins, as well as by 2,3-butanedione monoxime (**13**) has been shown to be responsible for the complexation of copper atoms that are present in the catalytic center of blue mussel PO [8]. Whether inhibition of blue mussel PO by DBHB (**1**) and by some of its derivatives is caused by direct complexation of copper ions in the active site of the enzyme or whether hemibastadins form a pre-Michaelis complex, which leads to a hindered substrate supply, as shown recently for the mushroom tyrosinase inhibitor tropolone [9], remain to be elucidated in future investigations.

## 3. Experimental Section

### 3.1. General Experimental Procedures

All reagents used in this study were purchased from commercial suppliers. Solvents for reactions and column chromatography were used at per analysis quality. MiliQ water and HiPerSolv CHROMANORM^®^ Methanol (VWR) were used for HPLC analysis and purification steps. Thin-layer chromatography (TLC) was performed using aluminum-backed plates coated with silica gel 60, F254 (Merck, Darmstadt, Germany), and compound spots were visualized by a UV lamp (LAMAG) at λ_max_ = 254 nm. Column chromatography was executed using silica gel (Macherey-Nagel, Silica 60 M, 0.04–0.063 mm). HPLC analysis was performed on a Dionex Ultimate 3000 System employing a Knauer VertexPlus Column (125 × 4 mm, Eurospher 100–10, C18). ESI mass spectra were recorded on a Thermoquest Finnigan LCQDeca connected to an Agilent 1100 Series LC. Preparative purification was performed on a Varian Prepstar connected to a Varian Prostar UV-detector. Semipreparative purification was accomplished on a Merck Hitachi system consisting of an L-7400 UV detector and an L-7100 pump connected with a Kipp&Zonen flatbed recorder with a Knauer VertexPlus C18 column (300 × 8 mm, Eurospher 100–10). All NMR spectra were recorded on a Bruker DRX 500 spectrometer (500 MHz ^1^H, Bruker, Billerica, MA, USA) and are presented in the Supporting Information.

### 3.2. Blue Mussel PO Inhibition Assay

PO activity was measured spectrophotometrically as described earlier [3]. The purified enzyme was incubated at 25 °C with 10 mM l-DOPA in 50 mM phosphate buffer of pH 6.8. PO activity was determined by monitoring the increase of absorbance at 475 nm. One unit of enzyme activity was defined as the amount of enzyme that catalyzes the formation of 1 μmol dopachrome per minute under the described experimental conditions. Hemibastadin congeners were added to the assay at concentrations of up to 50 μg/mL. In addition, the biocide TBT (10 μg/mL) was used as a positive standard. Aliquots of pure enzyme were incubated for 2 h with hemibastadin analogues, then the enzyme activity was recorded with l-DOPA or catechol (10 mM) as substrates. All assays were run in triplicate. The results are presented as the concentration of compounds that reduces enzyme velocity by 50% (IC_50_ values). IC_50_ values were calculated using MINITAB (Version 14).

### 3.3. Synthetic Procedures

Hemibastadin analogues were synthesized by an optimized and extended method, as described earlier [7,8] (Figure 1). Structures were confirmed via LC-ESI-MS and ^1^H-NMR (for spectra of new hemibastadin congeners see supporting information).

#### 3.3.1. l-Tyrosine-methyl Ester (**15**)

l-Tyrosine (**14**, 10.06 g, 55.53 mmol) was converted into the methyl ester (**15**) by dropwise addition of three equivalents thionyl chloride (SOCl_2_, 12.08 mL, 166.59 mmol) in methanol (100 mL) on ice. After complete addition of SOCl_2_, the suspension was heated under reflux for 12 h. The resulting solution was concentrated *in vacuo* and the pH was adjusted to 8 with NaHCO_3_, followed by exhaustive extraction with ethyl acetate. The organic phase was separated and dried over anhydrous magnesium sulfate. After solvent evaporation, 9.86 g of l-tyrosine-methyl ester (**15**) were obtained as a white, amorphous powder (50.53 mmol, 91.0%). Positive mode ESI-MS analysis revealed the pseudomolecular ion [M + H]^+^ at *m/z* 196.

#### 3.3.2. (*E*)-Methyl 2-(hydroxyimino)-3-(4-hydroxyphenyl)propanoate (**16**)

Oxidation of **15** to **16** was carried out according to a modified procedure of Boehlow *et al.* [10]. The ester **15** (8.59 g, 44 mmol) was dissolved in ethanol (100 mL) and stirred on ice. After the addition of H_2_O (76 mL), equimolar amounts of sodium tungstate dihydrate (Na_2_WO_4_·2H_2_O, 44 mmol) and H_2_O_2_ (30% aqueous solution, 44 mL), the solution was stirred until the color changed into pale yellow, and TLC analysis (SiO_2_, dichloromethane, ethyl acetate 3:1) revealed a complete turnover of the educt. After exhaustive extraction with ethyl acetate, the combined organic phases were washed with aqueous sodium hydrogen sulfite (2 × 100 mL) and H_2_O (2 × 100 mL) and dried over anhydrous magnesium sulfate. The solvent was removed *in vacuo* to yield a pale yellow powder. The crude product was further purified by column chromatography (SiO_2_, dichloromethane, ethyl acetate 3:1), and 7.09 g of pure **16** (33.90 mmol, 77.0%) were obtained as a nearly white amorphous powder. All spectral data were in accordance with previously reported values [10].

#### 3.3.3. (*E*)-Methyl 3-(3,5-dibromo-4-hydroxyphenyl)-2-(hydroxyimino)propanoate (**17**)

The ester **16** (1 g, 4.78 mmol) was dissolved in dimethylformamide (10 mL) and diluted with dichloromethane (60 mL). After cooling on crushed ice, a solution of bromine (11 mL 1M-Br_2_ in dichloromethane) was added dropwise during 20 min of stirring and cooling in the absence of light. TLC analysis was used to control complete bromination. Excessive bromine was reduced by the addition of aqueous 10% sodium hydrogen sulfite solution until the brown color was converted to pale yellow. The water phase was separated and extracted exhaustively with ethyl acetate. Both organic phases were washed with water, then combined and dried over anhydrous magnesium sulfate. After solvent evaporation, the resulting crude product was purified by column chromatography (SiO_2_, dichloromethane, ethyl acetate 5:1) to yield 1.53 g of pure **17** (4.17 mmol, 87.3%) as an amorphous white powder. All spectral data were in accordance with previously reported values [10].

#### 3.3.4. 5,5′-Dibromohemibastadin-1 (**1**) and Norbromohemibastadin-1 (**2**)

DBHB (**1**) and nobromohemibastadin-1 (**2**) were synthesized by replication of the protocol published earlier [8]. All spectral data were in accordance with previously reported values [8].

#### 3.3.5. Tyrosinyltyramine (**3**)

Tyrosinyltyramine (**3**) was synthesized by replication of the protocol published earlier, and all spectral data were in accordance with [7].

#### 3.3.6. Tetraiodo-norbromohemibastadin-1 (**4**)

Tetraiodo-norbromohemibastadin-1 (**4**) was synthesized by iodination of **2**, utilizing a modified method of Wada *et al.* [11]. Briefly, **2** (157.0 mg, 0.5 mmol) was dissolved in diluted sodium hydroxide solution (5 mL, pH 10), and H_2_O_2_ (30% aqueous solution, 0.12 mL) and iodine (I_2_, 152.0 mg, 0.6 mmol) were added. The solution was stirred at 55 °C for 3 h. Excessive iodine was reduced by the addition of aqueous 10% sodium hydrogen sulfite solution. After solvent evaporation *in vacuo*, the resulting crude product was purified by column chromatography (SiO_2_, hexan, ethyl acetate, 4:1), and pure **4** (3.7 mg, 4.5 µmol, 0.9%) was obtained. **4**: ^1^H (500 MHz, DMSO-*d*_6_) δ 11.90 (s, 1H), 9.40 (s, 1H), 9.35 (s, 1H), 8.02 (t, *J* = 5.9 Hz, 1H), 7.56 (s, 2H), 7.54 (s, 2H), 3.65 (s, 2H), 3.30–3.26 (m, 2H), 2.61 (t, *J* = 7.2 Hz, 2H); ESI-MS [M + H]^+^ *m*/*z* 818.7.

#### 3.3.7. Tetrachloro-norbromohemibastadin-1 (**5**)

To a solution of **2** (157.0 mg 0.5 mmol) in a mixture of tetrahydrofuran (3 mL) and isopropanol (1 mL), *N*-chlorosuccinimide (333.8 mg, 2.5 mmol) was added in portions, and the suspension was stirred at room temperature for 2 h. After solvent evaporation *in vacuo*, the crude product was purified via column chromatography (SiO_2_, dichloromethane, ethyl acetate 1:1), and semipreparative HPLC (gradient system of 0.1% trifluoro acetic acid and methanol) was conducted to obtain pure **5** as a yellow solid (3.7 mg, 8.2 µmol, 1.6%). **5**: ^1^H (500 MHz, DMSO-*d*_6_) δ 11.90 (s, 1H), 9.93 (s, 1H), 9.85 (s, 1H), 7.99 (t, *J* = 5.8 Hz, 1H), 7.15 (s, 2H), 7.14 (s, 2H) 3.69 (s, 2H), 3.33 (m, 2H), 2.67 (t, *J* = 7.0 Hz, 2H); ESI-MS [M + H]^+^ 453.3, [M − H]^−^ *m*/*z* 451.3.

#### 3.3.8. Tri-*O*-methyl-5,5′-dibromohemibastadin-1 (**6**)

Methylation of both phenolic hydroxyls and of the oxime function of **1** (189 mg, 0.3 mmol) was achieved in acetone (6 mL) by the addition of potassium carbonate (207.0 mg, 1.5 mmol) and iodomethane (63.9 mg, 0.45 mmol), heating under reflux for 8 h and additional stirring at RT for 12 h. After solvent evaporation and subsequent purification of the crude product via semipreparative HPLC (gradient system of 0.1% trifluoro acetic acid and methanol), pure **6** (46.0 mg, 0.07 mmol, 23.0%) was obtained as a white powder. **6**: ^1^H (500 MHz, DMSO-*d*_6_) δ 8.27 (t, *J* = 5.8 Hz, 1H), 7.47 (s, 2H), 7.42 (s, 2H), 3.97 (s, 3H), 3.76–3.75 (m, 8H), 3.35 (t, *J* = 6.9 Hz, 2H), 2.73 (t, *J* = 6.9 Hz, 1H); ESI-MS [M + H]^+^ *m*/*z* 672.9.

#### 3.3.9. Amides Resulting from Liquid Primary Amines (**7**–**10**)

For the preparation of Compounds **7**–**10**, four aliquots of **17** (200 mg, 0.96 mmol) were dissolved in ten equivalents (9.6 mmol) of a liquid primary amine (for **7**: 1.11 mL cyclohexylamine; for **8**: 0.82 mL isobutylamine; for **9**: 1.26 mL *n*-hexylamine; for **10**: 1.21 mL phenethylamine), respectively, and stirred at 60 °C for 12 h in an open flask. For the general work-up, the crude products (**7**–**10**) were diluted with ethyl acetate (50 mL) and aqueous 10% HCl (10 mL). After separation, the aqueous phase was extracted exhaustively with ethyl acetate. The organic phases were combined and dried over anhydrous magnesium sulfate. After solvent evaporation, the resulting solids were further purified by column chromatography (SiO_2_, dichloromethane, ethyl acetate 3:1) to obtain the pure compounds:

*N*-cyclohexyl-3-(3,5-dibromo-4-hydroxyphenyl)-2-(2-hydroxyimino)-propanamide (**7**): 371.8 mg (89.2%), ^1^H-NMR (500 MHz, DMSO-*d*_6_) δ 11.83 (s, 1H), 9.77 (s, 1H), 7.71 (d, *J* = 8.0 Hz, 1H), 7.35 (s, 2H), 3.69 (s, 2H), 3.66–3.53 (m, 1H), 1.72–1.62 (m, 3H), 1.55 (d, *J* = 11.0 Hz, 1H), 1.32–1.18 (m, 5H), 1.13–1.02 (m, 2H); ESI-MS [M + H]^+^ *m*/*z* 435.1.

*N*-(2-methyl-propyl)-3-(3,5-dibromo-4-hydroxyphenyl)-2-(2-hydroxyimino)-propanamide (**8**): 385.5 mg (98.4%), ^1^H (500 MHz, DMSO-*d*_6_) δ 11.87 (s, 1H), 9.77 (s, 1H), 8.00 (t, *J* = 6.6 Hz, 1H), 7.34 (s, 2H), 3.71 (s, 2H), 2.95 (t, *J* = 6.6 Hz, 2H), 1.82–1.69 (1H, m), 0.80 (d, *J* = 6.6 Hz, 6H); ESI-MS [M + H]^+^ *m*/*z* 409.0.

*N*-hexyl-3-(3,5-dibromo-4-hydroxyphenyl)-2-(2-hydroxyimino)-propanamide (**9**): 383.9 mg (91.7%), ^1^H (500 MHz, DMSO-*d*_6_) δ 11.86 (s, 1H), 9.76 (s, 1H), 7.98 (t, *J* = 6.6 Hz, 1H), 7.34 (s, 2H), 3.70 (s, 2H), 3.11 (dd, 2H, *J* = 6.6, 6.9 Hz), 1.40 (t, 2H, *J* = 6.9 Hz), 0.83 (t, *J* = 6.5 Hz, 3H); ESI-MS [M + H]^+^ *m*/*z* 436.9.

*N*-2-phenylethyl-3-(3,5-dibromo-4-hydroxyphenyl)-2-(2-hydroxyimino)-propanamide (**10**): 409.0 mg (93.4%), ^1^H (500 MHz, DMSO-*d*_6_) δ 11.89 (s, 1H), 9.74 (s, 1H), 7.98 (t, *J* = 5.9 Hz, 1H), 7.34 (s, 2H), 7.25 (t, *J* = 7.4 Hz, 2H), 7.20–7.14 (m, 3H), 3.70 (s, 2H), 3.37 (dt, *J* = 5.9, 7.4 Hz, 2H), 2.75 (t, *J* = 7.4 Hz, 2H); ESI-MS [M + H]^+^ *m*/*z* 457.2.

#### 3.3.10. Amides Resulting from Solid Primary Amines (**11**, **12**)

Compounds **11** and **12** were obtained by triturating two aliquots of **17** (200 mg, 0.96 mmol) with the corresponding solid primary amine (each 2.11 mmol; for **11**: 234.5 mg histamine; for **12**: 338.1 mg tryptamine), adding dimethylformamide (3 mL) and melting at 130 °C for 30 min in an open flask. The resulting crude products were purified utilizing preparative HPLC (gradient system of 0.1% trifluoro acetic acid and methanol) to obtain the pure compounds:

*N*-[2-(4-imidazolyl)-ethyl]-3-(3,5-dibromo-4-hydroxyphenyl)-2-(2-hydroxyimino)-propanamide (**11**): 274.5 mg (64.1%), ^1^H (500 MHz, DMSO-*d*_6_) δ 14.27 (s, 1H), 11.98 (s, 1H), 9.81 (s, 1H), 8.96 (s, 1H), 8.21 (t, *J* = 6.0 Hz, 1H), 7.41 (s, 1H), 7.32 (s, 1H), 3.68 (s, 2H), 3.44 (dt, *J* = 6.0, 6.9 Hz, 2H), 2.83 (t, *J* = 6.9 Hz, 2H); ESI-MS [M + H]^+^ *m*/*z* 447.2.

*N*-[2-(3-indolyl)-ethyl]-3-(3,5-dibromo-4-hydroxyphenyl)-2-(2-hydroxyimino)-propanamide (**12**): 274.8 mg (57.8%), ^1^H (500 MHz, DMSO-*d*_6_) δ 11.92 (s, 1H), 10.79 (s, 1H), 9.78 (s, 1H), 8.06 (t, *J* = 5.9 Hz, 1H), 7.54 (d, *J* = 7.8 Hz, 1H), 7.37 (s, 2H), 7.33 (d, *J* = 8.0 Hz, 1H), 7.14 (s, 1H), 7.06 (dd, *J* = 7.5, 7.8 Hz, 1H), 6.96 (dd, *J* = 7.5, 8.0 Hz, 1H), 3.72 (s, 2H), 3.43 (m, 2H, overlapped with solvent signal), 2.85 (t, *J* = 7.6 Hz, 2H); ESI-MS [M + H]^+^ *m*/*z* 496.2.

## 4. Conclusions

In conclusion, among all synthetic analogues analyzed in this study, the natural product-like DBHB (**1**), which features all of the structural elements that are present in the sponge-derived hemibastadin and bastadin derivatives, showed the strongest inhibition of blue mussel PO. It is known that the enzyme inhibitory activity of DBHB (**1**) is paralleled by its strong antifouling activity, as demonstrated in experiments using barnacle larvae [7]. Naturally-occurring hemibastadins and bastadins show likewise strong anti-fouling activity against barnacle larvae [7]. Even though the latter had so far not been evaluated with regard to the inhibition of blue mussel PO, their close structural similarity to DBHB (**1**) strongly suggests that they will share the inhibitory activity of **1**. Based on the comparative investigations carried out in this study, it appears that natural selection has resulted in the accumulation of bastadin-like anti-fouling metabolites in the sponge. Since the preparation of DBHB (**1**) is comparatively simple, this synthetic compound is at the moment the most promising representative of bastadin-like compounds with regard to the inhibition of blue mussel PO.

## Supplementary Files

Supplementary File 1
