# Supplementary material for: SAR of Sponge-Inspired Hemibastadin Congeners Inhibiting Blue Mussel PhenolOxidase
Source: Mar Drugs. 2015 May 15;13(5):3061–71. doi: 10.3390/md13053061 (PMC4446618; doi:10.3390/md13053061)
Supplement: Supplementary File 1 [file marinedrugs-13-03061-s001.pdf]

Supplementary Information

Figure S1. ^{1}H-NMR spectrum of tetraiodo-norbromohemibastadin-1 (**4**)
Figure S2. ^{1}H-NMR spectrum of tetrachloro-norbromohemibastadin-1 (**5**)
Figure S3. ^{1}H-NMR spectrum of trimethoxy-5,5'-dibromohemibastadin-1 (**6**)
Figure S4. ^{1}H-NMR spectrum of *N*-cyclohexyl-3-(3,5-dibromo-4-hydroxyphenyl)-2-(2-hydroxyimino)-propanamide (**7**)
Figure S5. ^{1}H-NMR spectrum of *N*-(2-methyl-propyl)-3-(3,5-dibromo-4-hydroxyphenyl)-2-(2-hydroxyimino)-propanamide (**8**)
Figure S6. ^{1}H-NMR spectrum of *N*-hexyl-3-(3,5-dibromo-4-hydroxyphenyl)-2-(2-hydroxyimino)-propanamide (**9**)
Figure S7. ^{1}H-NMR spectrum of *N*-2-phenylethyl-3-(3,5-dibromo-4-hydroxyphenyl)-2-(2-hydroxyimino)-propanamide (**10**)
Figure S8. ^{1}H-NMR spectrum of *N*-[2-(4-imidazolyl)-ethyl]-3-(3,5-dibromo-4-hydroxyphenyl)-2-(2-hydroxyimino)-propanamide (**11**)
Figure S9. ^{1}H-NMR spectrum of *N*-[2-(3-indolyl)-ethyl]-3-(3,5-dibromo-4-hydroxyphenyl)-2-(2-hydroxyimino)-propanamide (**12**)

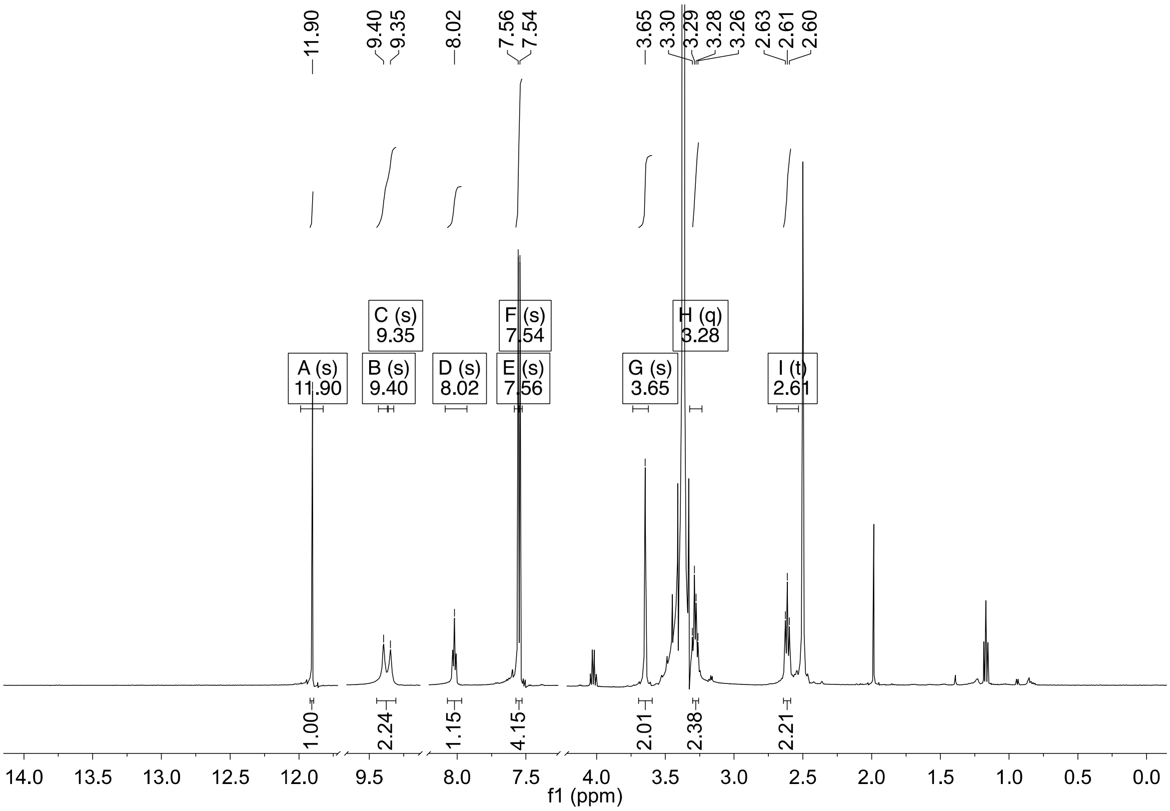

Figure S1. ^{1}H-NMR spectrum of tetraiodo-norbromohemibastadin-1 (**4**).

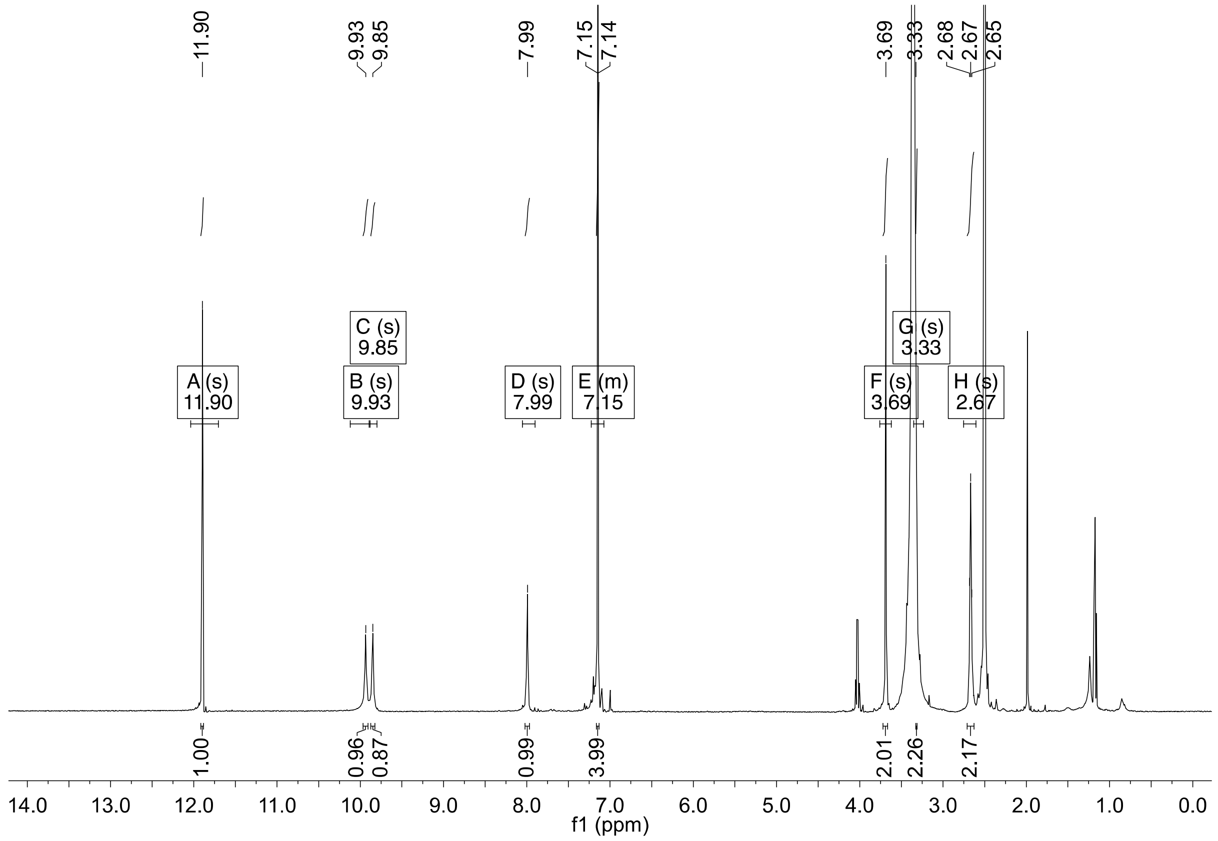

Figure S2. ^{1}H-NMR spectrum of tetrachloro-norbromohemibastadin-1 (**5**).

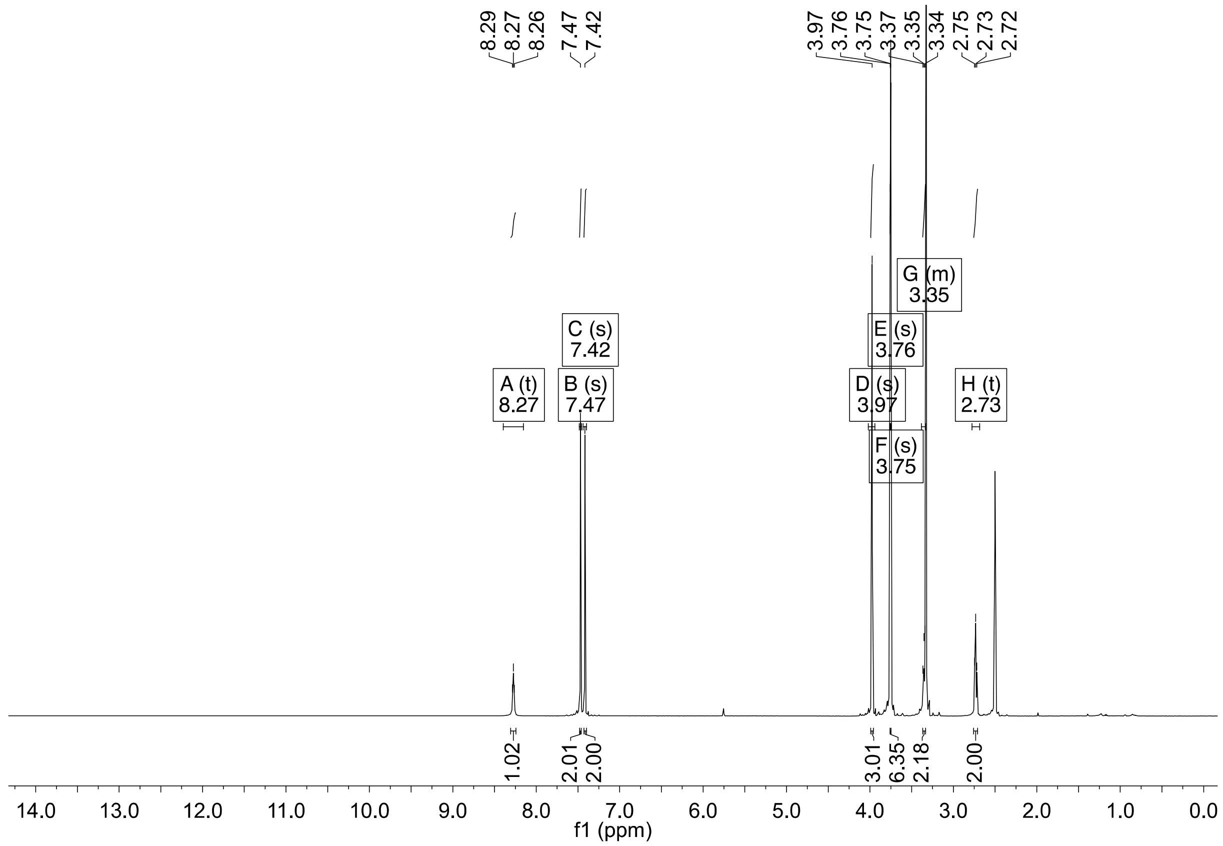

Figure S3. ^{1}H-NMR spectrum of trimethoxy-5,5′-dibromohemibastadin-1 (**6**).

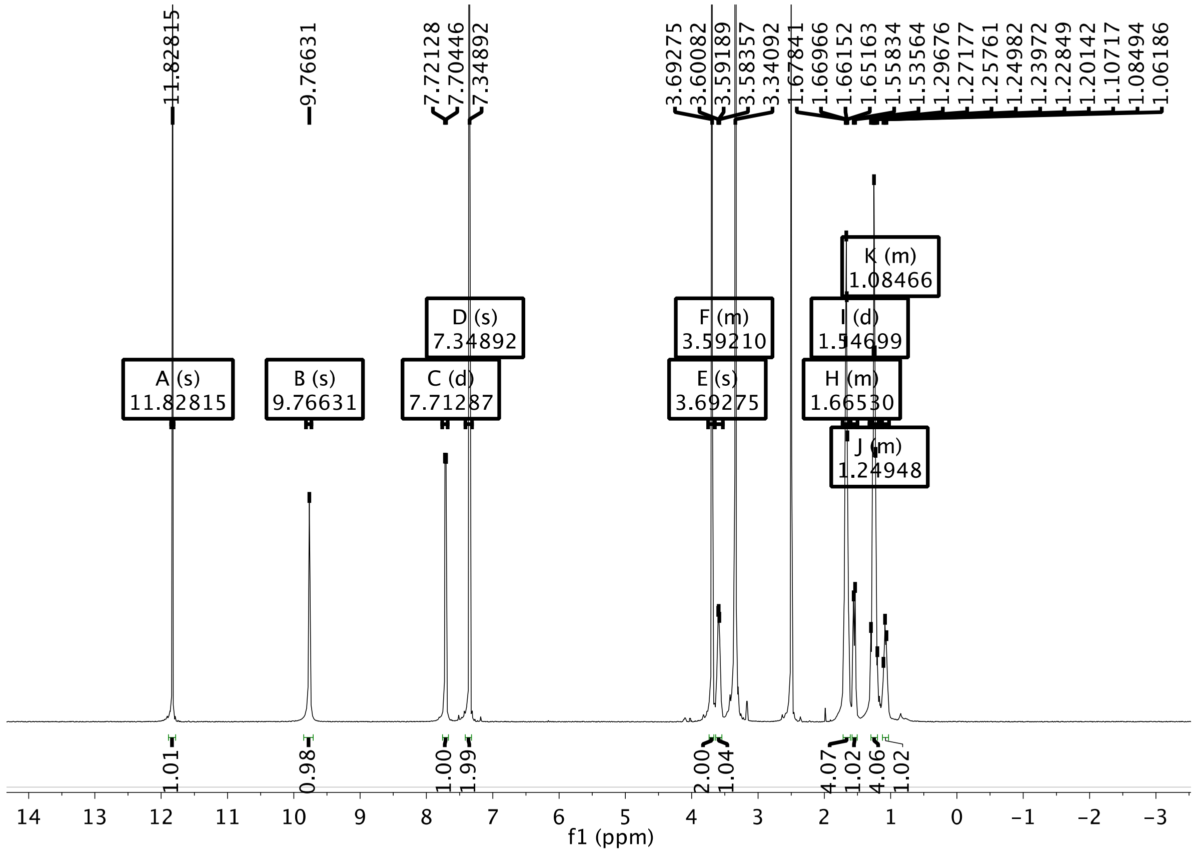

Figure S4. ^{1}H-NMR spectrum of *N*-cyclohexyl-3-(3,5-dibromo-4-hydroxyphenyl)-2-(2-hydroxyimino)-propanamide (**7**).

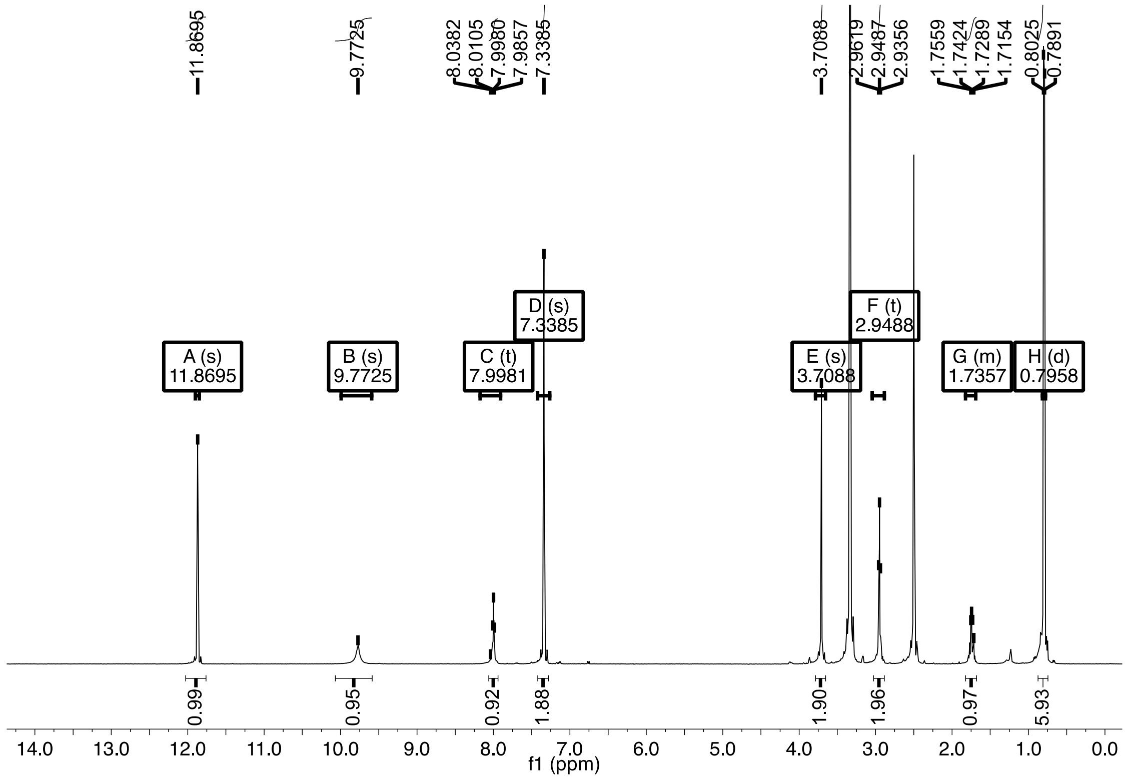

Figure S5. ^{1}H-NMR spectrum of *N*-(2-methyl-propyl)-3-(3,5-dibromo-4-hydroxyphenyl)-2-(2-hydroxyimino)-propanamide (**8**).

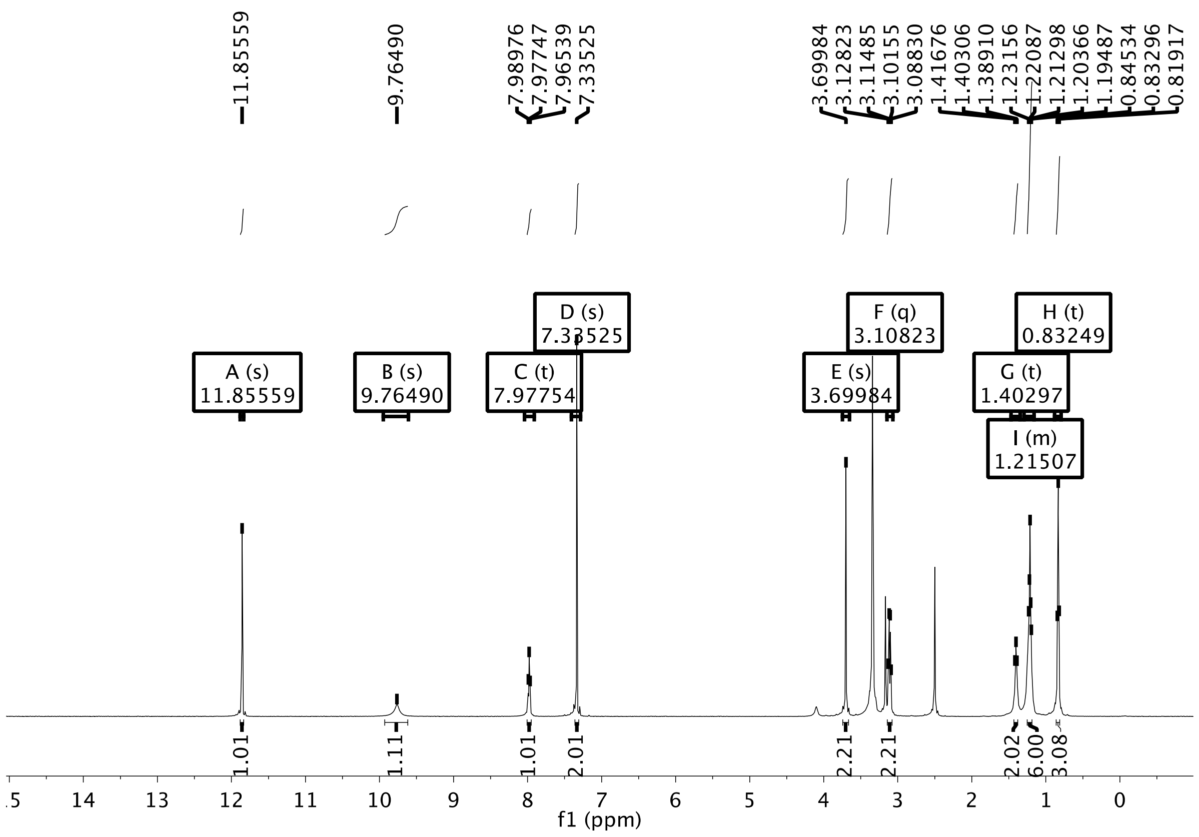

Figure S6. ^{1}H-NMR spectrum of *N*-hexyl-3-(3,5-dibromo-4-hydroxyphenyl)-2-(2-hydroxyimino)-propanamide (**9**).

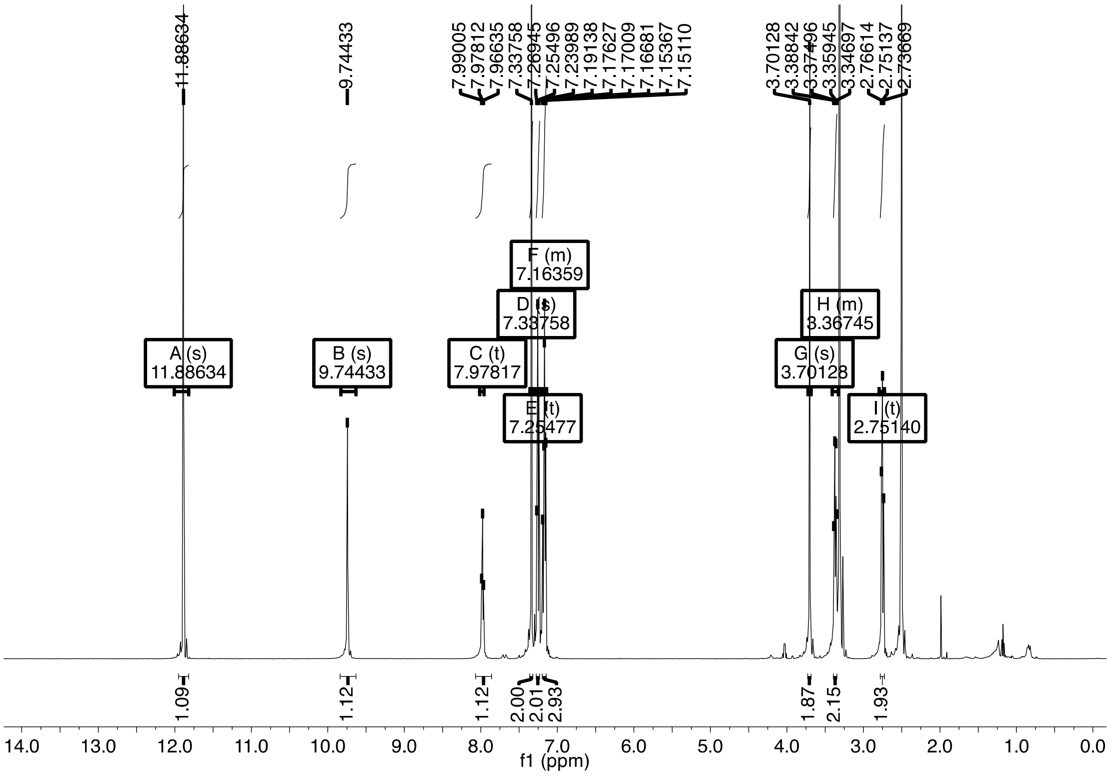

Figure S7. ^{1}H-NMR spectrum of *N*-2-phenylethyl-3-(3,5-dibromo-4-hydroxyphenyl)-2-(2-hydroxyimino)-propanamide (**10**).

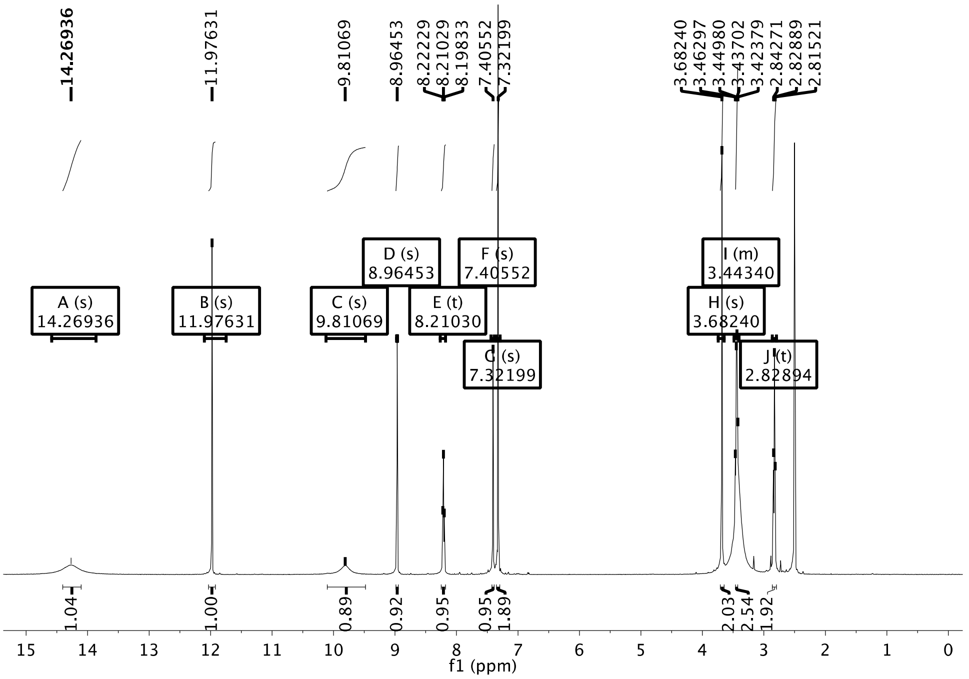

Figure S8. ^{1}H-NMR spectrum of *N*-[2-(4-imidazolyl)-ethyl]-3-(3,5-dibromo-4-hydroxyphenyl)-2-(2-hydroxyimino)-propanamide (**11**).

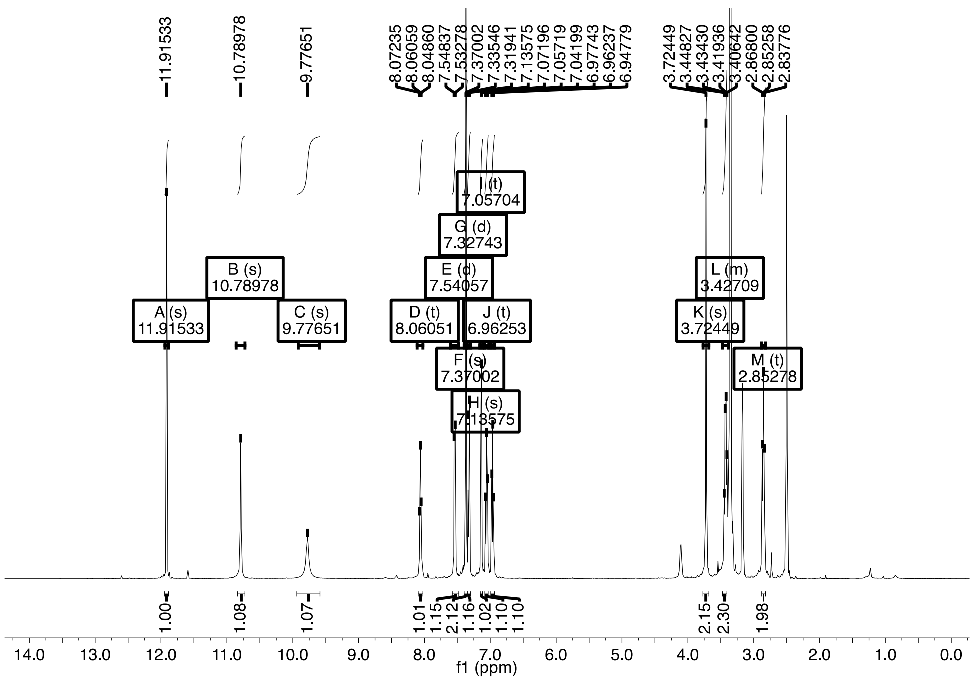

Figure S9. ^{1}H-NMR spectrum of *N*-[2-(3-indolyl)-ethyl]-3-(3,5-dibromo-4-hydroxyphenyl)-2-(2-hydroxyimino)-propanamide (**12**).

© 2015 by the authors; licensee MDPI, Basel, Switzerland. This article is an open access article distributed under the terms and conditions of the Creative Commons Attribution license (http://creativecommons.org/licenses/by/4.0/).
